# Adapted Pechini method to prepare DSA type electrodes of RuO_2_-ZrO_2_ doped with Sb_2_O_5_ over titanium plates

**DOI:** 10.1016/j.mex.2018.11.020

**Published:** 2018-11-30

**Authors:** Francisca A. Rodríguez, Eligio P. Rivero, Ignacio González

**Affiliations:** a Departamento de Ingeniería y Tecnología, Universidad Nacional Autónoma de México, Facultad de Estudios Superiores Cuautitlán, Av. 1o de Mayo, Cuautitlán Izcalli, Estado de México 54740, Mexico; b Departamento de Química, Universidad Autónoma Metropolitana-Iztapalapa, San Rafael Atlixco 186, México D. F. 09340, Mexico

**Keywords:** Pechini method adapted for the preparation of metal oxides over titanium plates, Dimensionally stable anode, Ruthenium oxide RuO_2_, Zirconium oxide ZrO_2_, Antimony oxide Sb_2_O_5_, Pretreatment of the titanium, Polymer mixture, Metallic precursors, Thermal method

## Abstract

This paper describes a thermal method to obtain metal oxides on a titanium substrate surface. This adapted Pechini method is a versatile, easy to handle and scalable technique to obtain electrodes for industrial uses, such as Dimensionally Stable Anodes (DSA). This method has advantages over other thermal methods like dip coating or sputtering, as it needs a smaller amount of polymeric mixture than dip coating method to cover the same area and is less expensive than sputtering method. The thermal method described herein to prepare DSA type electrodes of RuO_2_-ZrO_2_ doped with Sb_2_O_5_ over titanium plates needs no sophisticated equipment as spray pyrolysis technique does; a muffle, ultrasonic equipment, and a hot plate magnetic stirrer are the principal apparatus necessary to carry out the adapted Pechini method. On the other hand, this method allows metal oxides to disperse homogeneously. The cyclic voltammograms showed the stability of DSA, and the accelerated life test allowed establishing its useful life (18.18 years) at a current density of 10 mA cm^−2^.

## Graphical abstract

**Figure:**
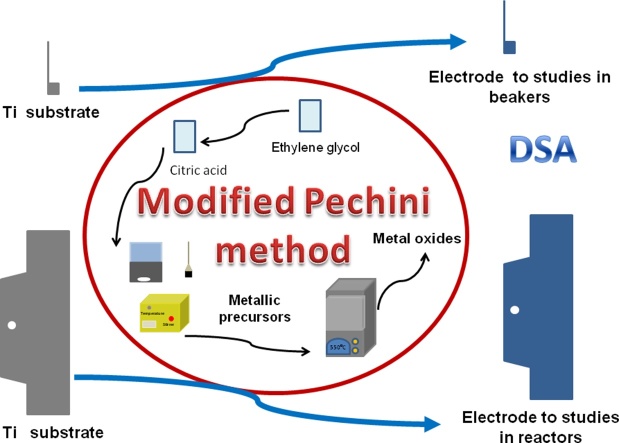

**Specifications Table**

**Table:** 

Subject area	*Select one of the following:*
	*Chemical Engineering*
More specific subject area	*Preparation of new materials*
Method name	*Pechini method adapted for the preparation of metal oxides over titanium plates*
Name and reference of original method	*Method of preparing lead and alkaline earth titanates and niobates and coating method using the same to form a capacitor.* *Pechini, M.P. (1967) Method of Preparing Lead and Alkaline Earth Titanates and Niobates and Coating Method Using the Same to Form a Capacitor. US Patent No.* 3330697.
Resource availability	*RuCl_3_, ZrO(NO_3_)_2_·H_2_O, SbCl_3_, ethylene glicol, citric acid, a muffle, ultrasonic equipment, and a hot plate magnetic stirrer.*

## Method details

The preparation of DSA type electrodes by this method consists of forming polyester from the reaction between ethylene glycol and citric acid, which allows dispersing the metals in a homogeneous way. Subsequently, by means of a thermal treatment, the organic matter is calcined, leaving the metal oxides supported on the Ti substrate. The method described in this work is an adaptation of the Pechini method [1] (developed to prepare powders of metal oxides as in others modified Pechini methods [2,3]) that can be used for the preparation of DSA type electrodes of metal oxides over titanium surfaces; the method includes a pretreatment of the titanium plates to obtain durable coatings.

**Fig. 1:**
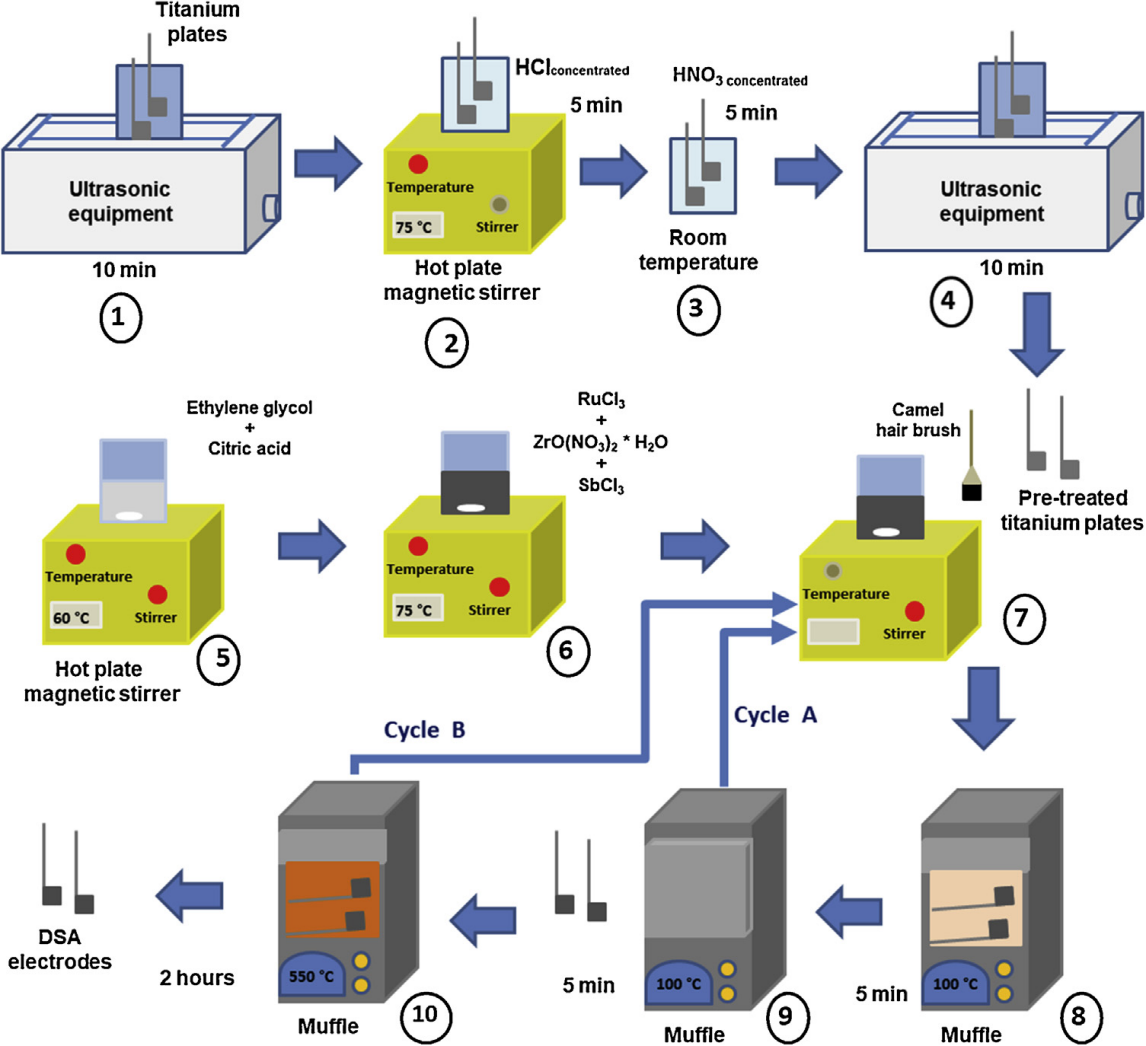
Flow chart of DSA electrode preparation using the modified Pechini method.

The Ti/RuO_2_-ZrO_2_ doped with Sb_2_O_5_ DSA electrode was prepared by the adapted Pechini method, whose steps are shown in Fig. 1. First, the titanium plates (1 × 1 cm) were given pretreatment consisting of 10-min cleaning in a Cole-Parmer 88920 ultrasonic unit (step 1), 5-min immersion in concentrated HCl at 75 °C (step 2), 5-min immersion in concentrated HNO_3_ at room temperature (step 3) to remove chlorides from the titanium surface, and finally, 10-min ultrasonic cleaning (step 4).

The reagents necessary to prepare the polymeric blend to ternary mixed metal electrode (Ti/RuO_2_-ZrO_2_ doped with Sb_2_O_5_) are: metal precursors and molar ratios employed, RuCl_3_ (0.0296), ZrO(NO_3_)_2_·H_2_O (0.0296), SbCl_3_ (0.0004), the polymeric precursor and molar ratios employed, ethylene glycol (16), citric acid (0.12), all reagents were analytical grade and the solutions were prepared with deionized water of 1 × 10^−6^ S cm^−1^ conductivity. If a different metal oxide is required, the precursor reagent of the metal of interest can be added. In this way we can adapt the method to obtain electrodes with a metallic oxide, a mixture of binary, ternary and even quaternary metal oxides.

In the preparation of the polymer blend, ethylene glycol was heated to 60 °C, then citric acid was added until a homogenous mixture was obtained (with constant stirring) (step 5). The temperature was increased to 75 °C and RuCl_3_ was added little by little in such a way that it took 20 min to complete the addition of 0.0669 g RuCl_3_; once all RuCl_3_ was dissolved, the other metal precursors were added one by one until a homogeneous solution was obtained, and finally maintained at 75 °C with constant stirring for 30 min (step 6). Lastly, the blend was allowed to cool to room temperature. Then the titanium plates were coated with the polymer mixture, using a fine hair brush, such as camel hair (step 7), and heated in a Thermolyne Type 1500 muffle at 100 °C for 5 min (step 8); subsequently the plates were allowed to cool for 5 min (step 9). Steps 7, 8 and 9 (cycle A) were performed 8 times to later activate the electrodes in the muffle at 550 °C for 1 h (step 10) and finish a complete cycle (cycle B) with 8 layers of metal oxides on the titanium surface. The cycle B described above (steps 7, 8 and 9 performed 8 times plus step 10) was also performed 8 times to finally finish with 64 layers of metal oxides on the surface of the titanium.

**Fig. 2:**
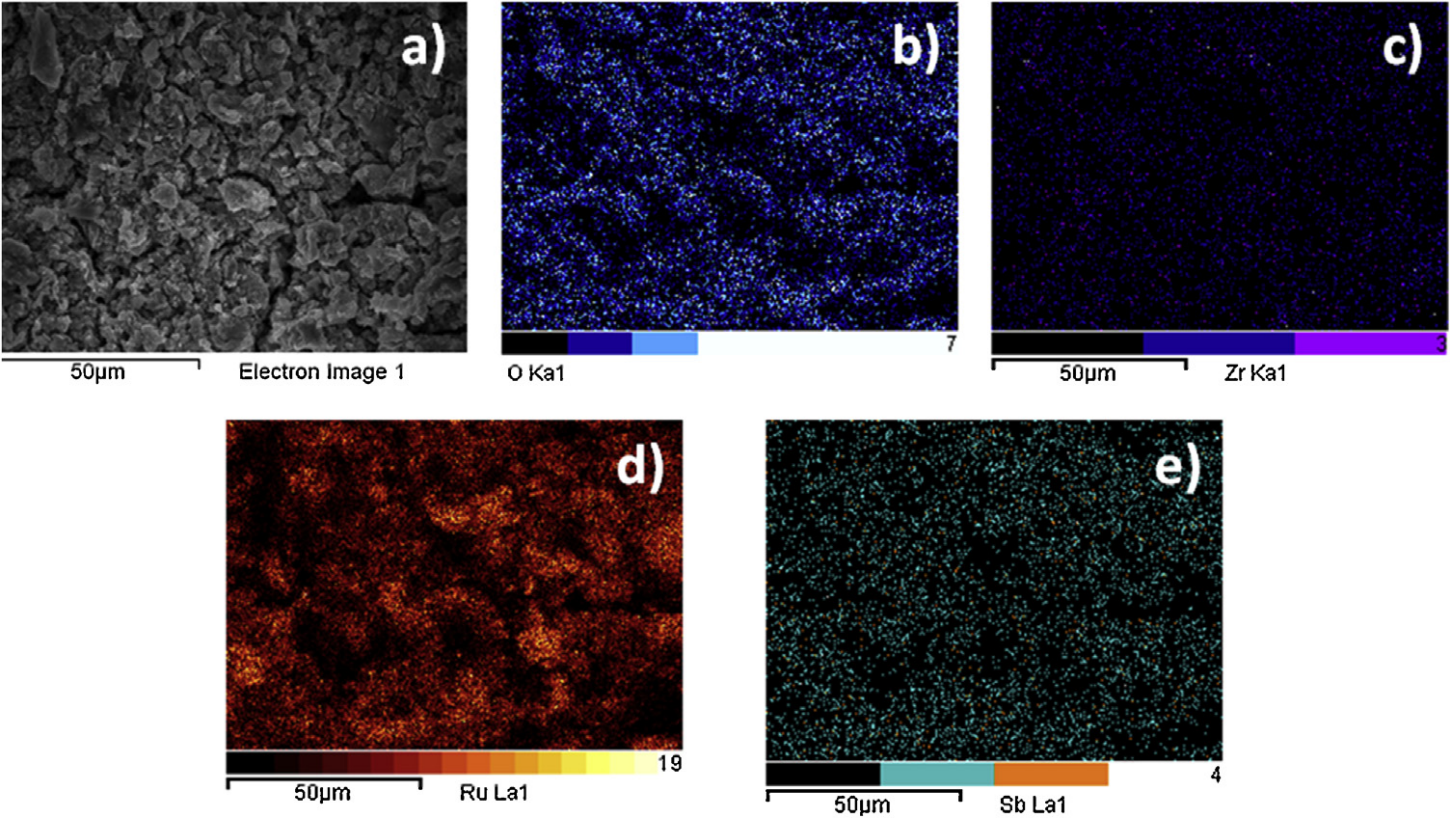
(a) SEM surface image of ternary mixture of DSA electrode. EDS surface element distribution, (b) Oxygen, (c) Zirconium, (d) Ruthenium, (e) Antimony.

The characterization of Ti/RuO_2_-ZrO_2_ doped with Sb_2_O_5_ electrode by X-ray diffraction and XPS was published by Rodriguez et al. [4], where the presence of metal oxides on the surface of the electrode was verified. Surface morphology of the deposited oxide films was analyzed using Scanning Electron Microscopy (SEM) and Energy Dispersive X-Ray Spectroscopy (EDS). Fig. 2 a) shows the morphology of the ternary mixture of DSA electrode, where the Pechini method generated a homogeneous surface, with cracking and porosity, which increases the active electrode surface. This has been attributed to the participation of ZrO_2_ as dispersing agent and Sb_2_O_5_ as dopant in the oxide mixture. EDS surface images exhibit a uniform dispersion of the analyzed elements, Fig. 2 b)–e).

**Fig. 3:**
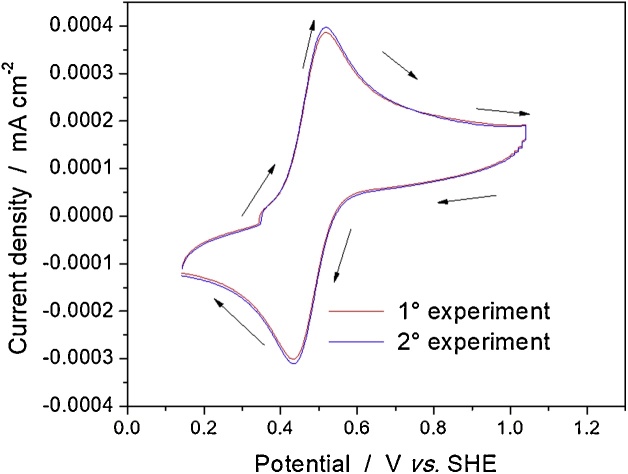
Cyclic voltammograms (20 mV s^−1^), Ti/RuO_2_-ZrO_2_ doped with Sb_2_O_5_ electrode. 50 mL 4 mM K_4_Fe(CN)_6_ and 1 M KNO_3_.

The stability of Ti/RuO_2_-ZrO_2_ doped with Sb_2_O_5_ electrode was tested with cyclic voltammetry at 20 mV s^−1^ in a potential range of 0.5−1 V *vs.* SHE. The system used consisted of a three-electrode cell, where the anode was the ternary electrode, cathode was a graphite rod and the reference electrode was a saturated calomel electrode, and the electrolyte was a solution (50 mL) containing 4 mM K_4_Fe(CN)_6_ and 1 M NaNO_3_. Fig. 3 shows two cyclic voltammograms (20 mV s^−1^) that are very similar, which means that the properties of the oxides do not change with the applied potential.

**Fig. 4:**
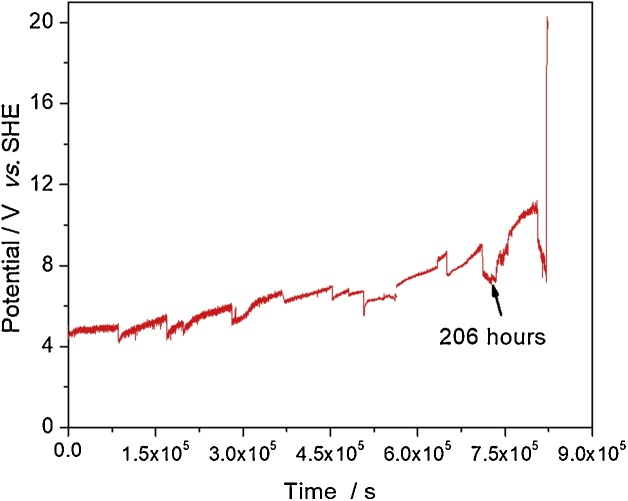
Behavior of the potential of a ternary mixture DSA electrode (Ti/RuO_2_-ZrO_2_ doped with Sb_2_O_5_) during accelerated life test.

Accelerated life test was performed in order to estimate the durability of the DSA electrode. A typical three-electrode cell was used; the DSA (Ti/RuO_2_-ZrO_2_ doped with Sb_2_O_5_) with 1 cm^2^ surface area was used as the working electrode and a graphite rod as the counter electrode, whereas the reference electrode was a saturated calomel electrode equipped with a Luggin capillary. 1 M NaCl was used as supporting electrolyte, and the cell temperature was controlled at about 25 °C. A power supply provided a constant anodic current density of 0.5 A cm^−2^. The solution was replaced each 24 h and the potential of the working electrode was periodically monitored. Fig. 4 exhibits the potential of DSA during the accelerated life test, which at 206 h shows an abrupt change related to a change in the surface of the electrode (detached metal oxides). Therefore, the electrode is no longer useful for what it was designed. Its useful life was estimated with the following equation [5]:

(1) $$\text{S}{\text{L}}_{\text{EA}}=\text{S}{\text{L}}_{\text{a}}{\left(\;\frac{{\text{j}}_{\text{a}}}{{\text{j}}_{\text{EA}}}\;\right)}^{1.7}$$

Where SL_EA_ and j_EA_ are service life and current density of electrolysis application, SL_a_ and j_a_ are the service life and current density under the accelerated life test conditions. The estimated useful life of the prepared electrode is 18.8 years, which is competitive for industries.
